# Patient Cognitive Impairment Associated With Greater Care Intensity During Medicare Home Health Care

**DOI:** 10.1093/geroni/igab046.708

**Published:** 2021-12-17

**Authors:** Julia Burgdorf, Kathy Bowles

**Affiliations:** 1 Center for Home Care Policy & Research, New York, New York, United States; 2 University of Pennsylvania School of Nursing, Philadelphia, Pennsylvania, United States

## Abstract

Medicare beneficiaries with cognitive impairment are more likely to access home health care than those without such impairment, and an estimated 1 in 3 Medicare home health patients has diagnosed dementia. However, recent changes to the Medicare home health payment system do not adjust for patients’ cognitive impairment. To the extent that cognitive impairment prompts higher intensity care, this could create a financial disincentive for providers serving this patient population. We draw on a nationally representative sample of 1,214 (weighted n=5,856,333) community-living Medicare beneficiaries who received home health care between 2011-2016. We measure care intensity by the number and type of visits received during an index home health care episode. We model care intensity as a function of patient cognitive impairment during the episode, measured via clinician reports in standardized patient assessment data. In propensity score adjusted, multivariable models holding all covariates at their means, home health patients with identified cognitive impairment received a significantly greater number of visits. During the index home health episode, cognitively impaired patients received an additional 2.82 total visits (95% CI: 1.32-4.31; p<0.001), 1.39 nursing visits (95% CI: 0.49-2.29; p=0.003), 0.72 physical therapy visits (95% CI: 0.06-1.39; p=0.03), and 0.60 occupational therapy visits (95% CI: 0.15-1.05; p=0.01). Findings suggest that recent changes to Medicare home health care reimbursement do not reflect the more intensive care needs of patients with cognitive impairment, and may threaten access to care for these individuals.

